# Regular use of low-dose of opioids after gastrointestinal surgery may lead to postoperative gastrointestinal tract dysfunction in children: a Chinese national regional health center experience sharing

**DOI:** 10.1186/s12876-023-02999-7

**Published:** 2023-10-31

**Authors:** Fangyu Dai, Rensen Zhang, Ruyu Deng, Guoyong Wang, Hongjie Guo, Chunbao Guo

**Affiliations:** 1 Department of Pediatrics, Chongqing health center for women and children, Chongqing, P.R. China; 2 Anesthesiology Class 1, Chongqing Medical University, 2020 Chongqing, P.R. China; 3 Department of Pediatric General Surgery, Children’s Hospital, Chongqing Medical University, Chongqing, P.R. China; 4 Department of Anesthesiology, Children’s Hospital of Chongqing Medical University, Chongqing, P.R. China; 5 Department of Respiratory Medicine, National Clinical Research Center for Child Health and Disorders, Ministry of Education Key Laboratory of Child Development and Disorders, Chongqing Key Laboratory of Pediatrics, Children’s Hospital of Chongqing Medical University, Chongqing, P.R. China; 6 Department of Pediatrics, Women and Children’s Hospital of Chongqing Medical University, Chongqing, P.R. China; 7 Department of Pediatric General Surgery, Women and Children’s Hospital of Chongqing Medical University, 120 Longshan Road, Yubei District, 401147 Chongqing, Chongqing, P.R. China

**Keywords:** Postoperative pain, Postoperative gastrointestinal tract dysfunction, Enhanced recovery after Surgery, Patient-controlled analgesia, Pediatrics, Meckel’s diverticulum

## Abstract

**Background:**

The need for pain management is increasing in pediatrics, but the side effects of overuse or abuse of analgesics can be harmful to children’s health and even life-threatening in severe cases.

**Methods:**

Patients who underwent resection of Meckel’s diverticulum at the Children’s Hospital of Chongqing Medical University from July 1, 2019, to July 1, 2022, were included in this study. Opioids were administered through patient-controlled analgesia (PCA). Based on the preoperative choices made by the legal guardians, patients were stratified into two groups: PCA Group (PCAG) and Non-PCA Group (NPCAG). Data pertaining to the clinical characteristics and prognoses of these patients were subsequently collected and analyzed to assess the impact of opioid administration.

**Results:**

In the study, a total of 126 patients were enrolled, with 72 allocated to the Patient-Controlled Analgesia Group (PCAG) and 54 to the Non-Patient-Controlled Analgesia Group (NPCAG). When compared to the NPCAG, the PCAG exhibited a longer duration of postoperative fasting (median 72 vs. 62 h, p = 0.044) and increased utilization of laxatives (12[16.7%] vs. 2[3.7%], p = 0.022). However, the PCAG also experienced higher incidences of intestinal stasis and abnormal intestinal dilation (13[18.1%] vs. 3[5.6%], p = 0.037). No statistically significant differences were observed in pain assessments at the conclusion of the surgical procedure (0 vs. 1[1.9%], p = 0.429) or within the first 24 h postoperatively (16[22.2%] vs. 18[33.3%], p = 0.164). Additionally, NPCAG patients did not necessitate increased administration of rescue analgesics (2[2.8%] vs. 4[7.4%], p = 0.432).

**Conclusions:**

The administration of opioids did not demonstrably ameliorate postoperative pain but was associated with a heightened incidence of postoperative gastrointestinal tract dysfunction. The retrospective nature of the current research should be considered and should be clarified further.

## Introduction

Current research and scientific literature substantiate that children, including premature infants and neonates, are capable of experiencing pain. Failure to adequately assess and manage pain in this demographic can result in a range of detrimental outcomes [1–3]. As the concept has evolved, pain has gained recognition as the fifth vital sign, accentuating its significance in clinical settings. The demand for effective pain management is escalating within pediatrics; however, the injudicious application of analgesics may pose risks, including life-threatening complications in extreme cases [4].

Opioids, commonly employed for analgesia, are not without drawbacks. Respiratory depression stands as the most acute adverse effect [5, 6],followed by others such as bradycardia, hypotension, and postoperative gastrointestinal tract dysfunction (PGID) [4, 7, 8]. The prudent and safe utilization of opioids in pediatric patients undergoing gastrointestinal surgeries—a setting where PGID is notably prevalent—requires ongoing investigation.

The paradigm of Enhanced Recovery After Surgery (ERAS) has garnered widespread acceptance, with minimally invasive procedures and expedited recovery increasingly being integrated into clinical practice. However, the dissemination of ERAS in the pediatric population has been relatively sluggish, owing to the specialized needs and considerations inherent to this demographic. Drawing upon empirical data from adult cases and successful pediatric implementations, we posit that ERAS holds potential for improving clinical outcomes in pediatric patients [9]. Our research team is currently investigating strategies to optimize the adoption of ERAS in pediatric gastrointestinal surgeries, with particular emphasis on the enhancement of postoperative gastrointestinal function.

Meckel’s diverticulum (MD) stands as the most prevalent congenital gastrointestinal anomaly, predominantly manifesting in pediatric patients. Symptomatic cases typically arise between the ages of 2 and 8 years. In pediatric medicine, the surgical excision of symptomatic MD is generally advised, although the removal of incidentally discovered MD continues to be a subject of debate [10–12].

To elucidate the impact of opioid use on intestinal function recovery, we have compiled clinical data from patients who underwent Meckel’s diverticulum resections at our institution over a three-year span for further investigation.

## Methods

### Patient selection

In this single-center, retrospective study conducted at Children’s Hospital of Chongqing Medical University, the inclusion criteria encompassed all pediatric patients between the ages of 1 and 18 years who underwent Meckel’s diverticulum resection between July 1, 2019, and July 1, 2022. This included both symptomatic and incidental cases of Meckel’s diverticulum. The patients were excluded if they met any of the following criteria: (1) Postoperative Ventilator Dependency Requiring ICU Admission, referring to those who could not be extubated after surgery and consequently needed transfer to the Intensive Care Unit for further management, including ongoing sedation and mechanical ventilation; (2) Co-Existing Severe Gastrointestinal Diseases Necessitating Concurrent Surgical Intervention, encompassing patients diagnosed with severe gastrointestinal conditions like biliary atresia, necrotizing enterocolitis, or congenital megacolon, who also required simultaneous surgical procedures; (3) Presence of Severe Postoperative Surgical Site Infections, confirmed by literature cited in Reference [13] ; (4) Discovery of Meckel’s Diverticulum During Exploratory Laparotomy in Patients with Multi-Organ Injuries, relating to individuals found to have multiple organ injuries including, but not limited to, Meckel’s diverticulum upon exploratory laparotomy; and (5) Incomplete or Missing Clinical Data, which included patients whose clinical data were either missing or incomplete, thus affecting the interpretability and integrity of the study’s outcomes.

### Patient-controlled analgesia (PCA)

PCA has emerged as the primary modality for postoperative analgesia in pediatric patients owing to its demonstrated efficacy and safety [14–16]. Opioids are administered continuously via an analgesic pump, the selection of which is determined preoperatively by the patient’s legal guardian. The administration of PCA is exclusively managed by the Department of Anesthesiology, and attending clinicians are not involved in this aspect of patient care. In instances where patients or their guardians declined the use of PCA, no conventional analgesic regimen was provided until the onset of breakthrough pain.

Based on preoperative choices, patients were segregated into two groups: those utilizing Patient-Controlled Analgesia (PCAG) and those opting for no Patient-Controlled Analgesia (NPCAG).

### Utilization of patient-controlled analgesia (PCA)

A hydromorphone solution was prepared at a concentration of 0.15 mg/kg, totaling a volume of 100 mL, based on the patient’s body weight. Postoperatively, this solution was administered intravenously at a rate of 2 mL/h. For breakthrough pain episodes, the infusion rate could be temporarily increased by 0.5 mL upon activation of the manual switch.

Given the heightened risk of respiratory depression associated with opioid use, particularly in pediatric populations, vigilant monitoring is paramount [6, 17]. Anesthesiologists assessed the patient’s vital signs immediately postoperatively and within the first 24 h following surgery. Nursing staff conducted serial observations of vital signs at 2-hour, 6-hour, 12-hour, and 24-hour intervals to monitor for any adverse drug reactions or episodes of breakthrough pain requiring intervention.

Should pain management prove ineffective in either the PCAG or NPCAG cohorts, rescue protocols were activated: (1) Initial administration of nonsteroidal anti-inflammatory drugs (NSAIDs) such as ibuprofen or acetaminophen. (2) In cases where pain persisted and exhibited no significant relief within 30 min, morphine was employed. (3) Fentanyl was introduced if pain continued to worsen.

### Study endpoints

The primary endpoint of this investigation was the restoration of postoperative gastrointestinal function and the potential for postoperative gastrointestinal tract dysfunction. Secondary endpoints encompassed the efficacy and side effects of opioid analgesics.

### Data collection parameters

Clinical data accrued included age, body weight, presence of blood in stools, length of postoperative hospital stay, etiology necessitating surgery, results of diverticulum pathological examinations, Visual Analogue Scale (VAS) scores at surgery conclusion and within 24 h postoperatively, instances of breakthrough pain and interim analgesic use, postoperative pain assessments, duration of postoperative fasting, additional fasting and gastrointestinal decompression requirements post-meal, frequency of laxative administration, and outcomes of postoperative imaging studies, including color ultrasound and X-rays, as well as reoperation rates and opioid-related side effects.

### Statistical analysis methods

Statistical analyses were executed utilizing the Statistical Package for the Social Sciences (SPSS) software, version 25.0. The Kolmogorov–Smirnov test ascertained data distribution within all groups. Normally distributed data were evaluated using the Student’s t-test and reported as mean ± standard deviation (SD). In contrast, non-normally distributed data were examined using the Mann–Whitney U-test and presented as median along with the interquartile range (IQR; 25th–75th percentiles). Categorical variables were assessed employing either the Chi-squared (χ^2^) test or Fisher’s exact test, as appropriate. All tests were two-tailed, and a P-value less than 0.05 was deemed statistically significant.

## Results

### Patient characteristics

**Fig. 1:**
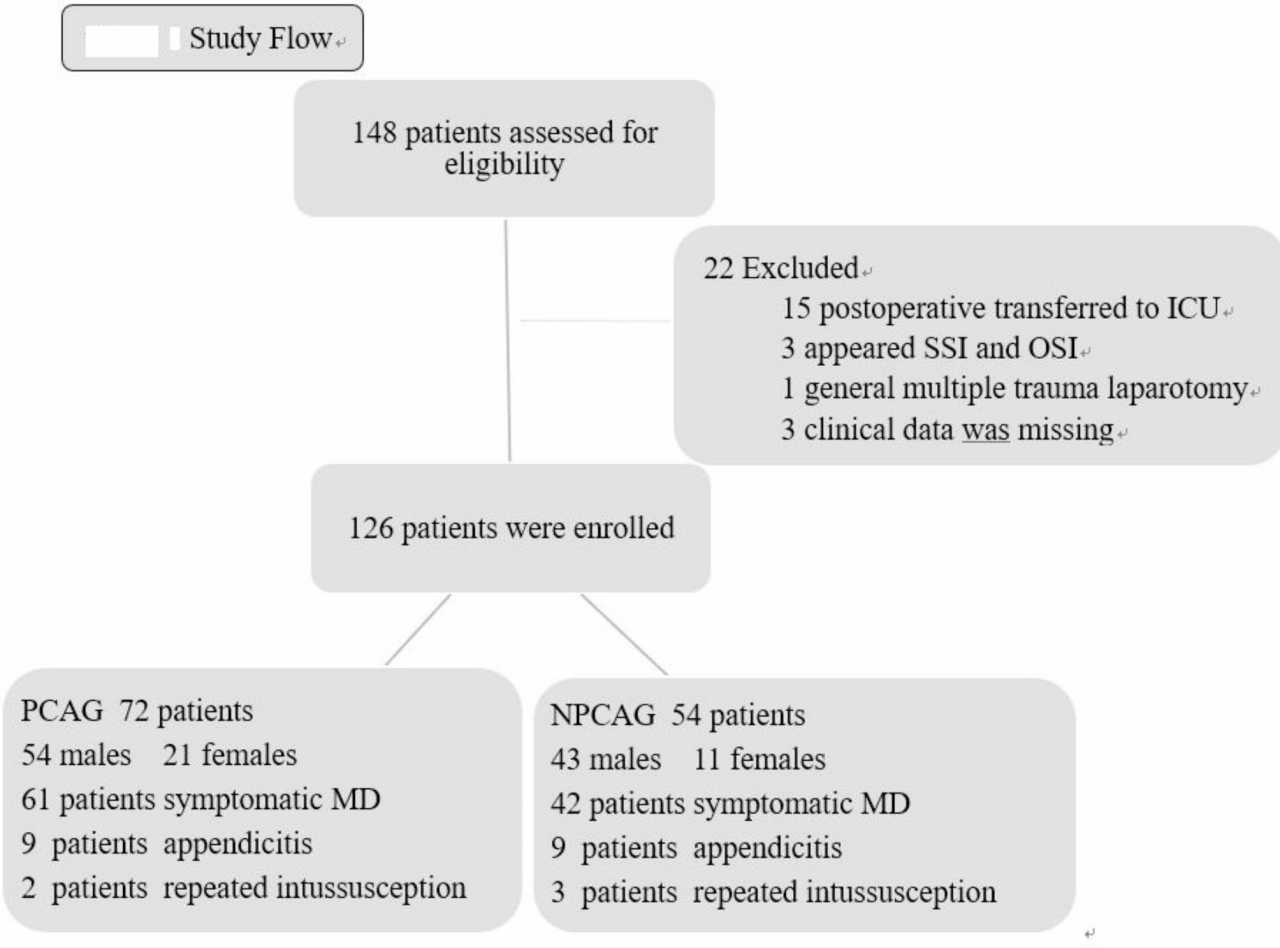
Flowchart for the patient enrollment

Of the 148 patients who underwent Meckel’s diverticular resection at our institution between July 1, 2019, and July 1, 2022, 22 were excluded from the study for various reasons. Specifically, 15 patients required postoperative intensive care unit (ICU) admission due to mechanical ventilation dependence, three presented with surgical site infections (SSIs) and organ space infections (OSIs), one patient was discovered to have a Meckel’s diverticulum during laparotomy for multiple traumas, and three had incomplete clinical data. Consequently, 126 patients were included in the clinical study, comprising 97 males and 29 females (a male-to-female ratio of 3.34:1). Among these, 103 underwent surgery for symptoms attributable to inflamed Meckel’s diverticulum, with bloody stools being the most prevalent symptom observed in 59 patients. Additionally, 18 patients were identified during routine intestinal exploration for appendicitis, and five were diagnosed during surgical interventions for recurrent intussusception. The study cohort was divided into 72 patients in the Patient Analgesic Preference Group (PAPG) and 54 in the No Patient Analgesic Preference Group (NPAPG) (Fig. 1).

No statistical differences were observed in baseline clinical characteristics—such as gender, age, body weight, recent disease course prior to admission, and reasons for surgery—between the two groups (Table 1).

**Table 1:** Comparison of Baseline Data Between PCAG and NPCAG

Baseline Data	PCAG (n = 72)	NPCAG (n = 54)	*P*
Sex Male, n (%) Female, n (%)	54(75) 18(25)	43(79.6) 11(20.4)	0.541
Age, median (IQR), month	95(47–131)	79(56–115)	0.470
Weight, median (IQR), Kg	24.8(15.0-39.6)	22.0(16.9–32.0)	0.450
The recent course of disease before admission, median (IQR), d	2(1–4)	2(1–3)	0.269
Causes of operation proved by intraoperative situation and pathological findings, n (%) 1. Treated by Michael’s diverticulitis 2. Found by appendicitis 3. Found by repeated intussusception	61(84.7) 9(12.5) 2(2.8)	42(77.8) 9(16.7) 3(5.6)	0.567

### Study end points

#### Recovery of gastrointestinal function

Postoperative gastrointestinal decompression was uniformly implemented following Meckel’s diverticular resection. Patients were initially administered a minimal volume of warm water; upon the absence of notable emesis, a gradual resumption of a liquid diet was initiated and the postoperative fasting duration was documented. The postoperative fasting duration was significantly longer for the Patient-Controlled Analgesia Group (PCAG) compared to the Non-Patient-Controlled Analgesia Group (NPCAG) (p = 0.044).

In cases of postoperative constipation, abdominal distension, or abdominal pain, laxative administration was necessitated. The incidence of requiring more than two laxative doses was significantly higher in the PCAG than in the NPCAG (p = 0.022).

The timing for postoperative assessment was contingent on patient recovery, generally occurring 4–5 days post-surgery. Delay or acceleration in this timing was patient-dependent. The postoperative assessment period was significantly protracted in the PCAG compared to the NPCAG (p = 0.035). Based on color ultrasound and X-ray evaluations, 13 patients in the PCAG exhibited postoperative intestinal stasis and abnormal dilation, whereas only 3 patients in the NPCAG exhibited these symptoms (p = 0.037).

No statistically significant differences were noted between the two groups in the incidence of postoperative intestinal wall thickening (p = 0.553), slowed intestinal peristalsis (p = 0.354), or minor peritoneal effusion (p = 0.132).

All patients were successfully discharged, and analysis revealed no significant variation in postoperative discharge times between the two groups (p = 0.977).

#### Efficacy evaluation of patient-controlled analgesia (PCA)

Upon regaining full consciousness postoperatively, a single patient in the Non-Patient-Controlled Analgesia Group (NPCAG) experienced mild pain, while the remainder of NPCAG and all patients in the Patient-Controlled Analgesia Group (PCAG) were pain-free. Anesthesiologists conducted Visual Analog Scale (VAS) assessments at random intervals within 24 h post-surgery. Of these, 16 patients in the PCAG (22.2%) and 18 patients in the NPCAG (33.3%) reported mild pain. No statistically significant differences were observed in the prevalence of mild pain immediately post-surgery (p = 0.429) or within the subsequent 24-hour period (p = 0.164) between the two groups.

Two patients in the PCAG and four in the NPCAG experienced breakthrough pain postoperatively. All improved following the administration of non-steroidal anti-inflammatory drugs (NSAIDs); statistical significance was not achieved between the groups (p = 0.432).

No incidents of respiratory depression were recorded in the PCAG, potentially attributed to the preoperative exclusion of patients with severe conditions necessitating continued ventilatory support. One patient in the PCAG experienced a febrile convulsion during hospitalization; a definitive correlation to analgesic pump side effects was not established.

Postoperative pain was not timely assessed in the NPCAG. In the PCAG, pain levels were documented at intervals of 2 h, 6 h, 12 h, and 24 h following PCA utilization. No patients reported moderate or severe pain, while mild pain was reported by 66 patients (91.7%) at 2 h, 65 patients (90.3%) at 6 h, 63 patients (87.5%) at 12 h, and 64 patients (88.9%) at 24 h (Table 2).

**Table 2:** Comparison of Clinical Characteristics Between PCAG and NPCAG

End Points	PCAG (n = 72)	NPCAG (n = 54)	*P*
Additional fasting and gastrointestinal decompression, n (%)	12(16.7)	4(7.4)	0.122
Analgesics use due to breakthrough pain, n (%)	2(2.8)	4(7.4)	0.432
Review time, median (IQR), d	5(4–5)	4(4–5)	0.035
Intestinal stasis and abnormal intestinal dilation, n (%) ^a^	13(18.1)	3(5.6)	0.037
Intestinal wall thickening, n (%) ^a^	4(5.6)	1(1.9)	0.553
Slowed intestinal peristalsis or anti-peristalsis, n (%) ^a^	3(4.2)	0(0)	0.354
Peritoneal effusion, n (%) ^a^	37(51.4)	35(64.8)	0.132
VAS at the end of surgery, n (%) ^b^	0(0)	1(1.9)	0.429
VAS within 24 h after surgery, n (%) ^b^	16(22.2)	18(33.3)	0.164
Use laxatives more than twice, n (%)	12(16.7)	2(3.7)	0.022
Analgesics use due to breakthrough pain, n (%)	2(2.8)	4(7.4)	0.432
Fasting time, median (IQR), h	72(60–84)	62(48–72)	0.044
Postoperative discharge time, median (IQR), d	7(6–7)	7(6–7)	0.977
Readmission due to intestinal obstruction, n (%)	5(6.9)	2(3.7)	0.694

a refers to postoperative abdominal color Doppler ultrasound or X-ray

b refers to mild pain

## Discussion

### Postoperative intestinal function

Opioids are currently the predominant choice for moderate to severe postoperative analgesia [18, 19]. Particularly in pediatric settings, the risk of opioid abuse is heightened due to a paucity of research and humanistic factors [17]. Given the numerous adverse effects that result from opioid overuse, recent literature recommends curtailing opioid use in favor of adopting a multimodal analgesia approach [20]. This approach is integral to Enhanced Recovery After Surgery (ERAS) [21], and can facilitate early postoperative mobilization, mitigate opioid-induced ileus, and expedite the transition to oral medications [22]. Utilizing NSAIDs, epidurals, and nerve blocks in a multimodal regimen can effectively reduce opioid consumption while maintaining analgesic quality [23–26].

Postoperative Gastrointestinal Dysfunction (PGID) is a frequent complication in clinical practice and is influenced by a myriad of factors [27]. The utilization of non-opioid analgesics such as NSAIDs, along with epidurals and nerve blocks, has gradually led to a reduced incidence of PGID [28, 29].

This retrospective study offers partial validation of these observations. Patients in the PCAG demonstrated elevated rates of gastrointestinal dysfunction; however, no significant differences were noted in terms of length of hospital stay or incidence of postoperative bowel obstruction between the two groups. Opioid administration during hospitalization may have contributed to these adverse experiences; hence, routine opioid use is not recommended.

In the NPCAG, analgesics were not administered routinely. NSAIDs were only employed when breakthrough pain was reported, effectively alleviating the discomfort. Existing literature suggests that pain can detrimentally impact intestinal perfusion, either directly via nociceptive stimuli [30], or indirectly by causing delays in postoperative activity and oral intake [30–32]. Although NSAIDs have been widely demonstrated to be safe in neonatal and pediatric populations [33, 34],, their routine use is cautioned against in colorectal surgeries due to an increased risk of anastomotic leakage [35–37][36–38].

Consequently, the potential role of non-opioid analgesics like intravenous acetaminophen in standard postoperative care warrants further research. It may be judicious to withhold postoperative analgesics altogether, utilizing NSAIDs or opioids solely for cases of breakthrough pain.

### Efficacy evaluation of PCA

Extant literature highlights respiratory depression as the most severe complication associated with opioid usage [5, 6]. Despite rigorous postoperative monitoring, we observed no instances of postoperative respiratory depression. This absence may be attributable to the exclusion criteria, which precluded patients unable to be weaned off ventilators and necessitating transfer to the intensive care unit for ongoing management.

Concurrent with postoperative surveillance, medical staff recorded the pain scores in children belonging to the PCAG. A majority exhibited mild pain, ranging between 87.5 and 91.7%. Regrettably, pain scores were not systematically recorded in the NPCAG, precluding a valid comparative analysis. Moreover, no significant differences in pain experiences were detected between the two cohorts, both immediately following the surgical procedure and during the postoperative assessments by the anesthesiologists. As such, the data suggests that opioid usage does not unequivocally enhance postoperative pain management, warranting further investigation through higher-quality studies.

### Limitations and future research implications

This study is marked by several limitations that concurrently outline avenues for future research. Firstly, the retrospective nature of our investigation inherently invites biases associated with this type of research design. To address this issue, future work should focus on prospective, randomized controlled trials for more accurate elucidation and validation of our observations. Secondly, the lack of systematically recorded pain scores in the NPCAG group restricted our ability to conduct a comprehensive comparative analysis. Subsequent research should ensure that pain scores are consistently recorded across all participant groups. Lastly, our study did not delve into how different doses or types of opioids specifically affect postoperative outcomes, mandating a more detailed examination in future studies. Given these limitations, there is a pressing need for further rigorous investigation into the potential role of non-opioid analgesics like intravenous acetaminophen in standard postoperative care. Additionally, future research should also focus on patient-centered long-term outcomes and quality of life.

## Conclusion

Our study underscores the imperative of revisiting prevalent opioid-based analgesia in pediatric gastrointestinal surgeries. Notably, opioids did not confer clear benefits in alleviating postoperative pain but increased the risk of Postoperative Gastrointestinal Tract Dysfunction (PGTD). This highlights the urgency to re-evaluate routine opioid administration, advising its reserved use only for breakthrough pain. As we confront a public health crisis around opioid misuse, these findings are timely. For future investigations, the emphasis should be on validating non-opioid alternatives and assessing their impact on long-term patient outcomes.

## Data Availability

The dataset analyzed during the current study are available from the corresponding author on reasonable request.

## Abbreviations

ERAS: Enhanced Recovery After Surgery
ICU: Intensive care unit
MD: Meckel’s diverticulum
NEC: Necrotizing enterocolitis
NPCAG: Non-Patient-controlled analgesia group
NSAIDs: Nonsteroidal antiinflammatory drugs
OSI: Organ space infection
PCA: Patient-controlled analgesia
PCAG: Patient-controlled analgesia group
PGID: Postoperative gastrointestinal tract dysfunction
SD: Standard deviation
SSI: Surgical site infection

## References

[1] Peters J (2005). Does neonatal Surgery lead to increased pain sensitivity in later childhood?. Pain.

[2] Bartocci M (2006). Pain activates cortical areas in the preterm newborn brain. Pain.

[3] Anand KJ, Runeson B, Jacobson B (2004). Gastric suction at birth associated with long-term risk for functional intestinal disorders in later life. J Pediatr.

[4] Martin DP (2014). The safety of prescribing opioids in pediatrics. Expert Opin Drug Saf.

[5] Niesters M (2013). Opioid-induced respiratory depression in paediatrics: a review of case reports. Br J Anaesth.

[6] Voepel-Lewis T (2008). The prevalence of and risk factors for adverse events in children receiving patient-controlled analgesia by proxy or patient-controlled analgesia after Surgery. Anesth Analg.

[7] Monitto CL (2011). The optimal dose of prophylactic intravenous naloxone in ameliorating opioid-induced side effects in children receiving intravenous patient-controlled analgesia morphine for moderate to severe pain: a dose finding study. Anesth Analg.

[8] Maxwell LG (2005). The effects of a small-dose naloxone infusion on opioid-induced side effects and analgesia in children and adolescents treated with intravenous patient-controlled analgesia: a double-blind, prospective, randomized, controlled study. Anesth Analg.

[9] Brindle ME (2019). Embracing change: the era for pediatric ERAS is here. Pediatr Surg Int.

[10] Hansen CC, Søreide K (2018). Systematic review of epidemiology, presentation, and management of Meckel’s diverticulum in the 21st century. Med (Baltim).

[11] Kuru S, Kismet K (2018). Meckel’s diverticulum: clinical features, diagnosis and management. Rev Esp Enferm Dig.

[12] Malik AA, Wani KA, Khaja AR (2010). Meckel’s diverticulum-revisited. Saudi J Gastroenterol.

[13] Kelly KN et al. Disease severity, not operative approach, drives organ space Infection after pediatric appendectomy. Ann Surg, 2014. 260(3): p. 466 – 71; discussion 472-3. 10.1097/SLA.0000000000000874 25115422

[14] Berde CB (1991). Patient-controlled analgesia in children and adolescents: a randomized, prospective comparison with intramuscular administration of morphine for postoperative analgesia. J Pediatr.

[15] Gaukroger PB, Tomkins DP, van der Walt JH (1988). Use of patient-controlled analgesia (PCA) in children. J Pediatr Surg.

[16] Anghelescu DL (2005). The safety of patient-controlled analgesia by proxy in pediatric oncology patients. Anesth Analg.

[17] Patient controlled analgesia by proxy. Sentin Event Alert, 2004(33): p. 1–2. 15612163

[18] Steyaert A, Lavand’Homme P (2013). Postoperative opioids: let us take responsibility for the possible consequences. Eur J Anaesthesiol.

[19] Thorén T (1989). Effects of epidural bupivacaine and epidural morphine on bowel function and pain after hysterectomy. Acta Anaesthesiol Scand.

[20] Chou R (2016). Management of Postoperative Pain: a clinical practice Guideline from the American Pain Society, the American Society of Regional Anesthesia and Pain Medicine, and the American Society of Anesthesiologists’ Committee on Regional Anesthesia, Executive Committee, and Administrative Council. J Pain.

[21] Kehlet H (1999). Acute pain control and accelerated postoperative surgical recovery. Surg Clin North Am.

[22] Ahmed J (2010). Predictors of length of stay in patients having elective colorectal Surgery within an enhanced recovery protocol. Int J Surg.

[23] Rømsing J (2005). Reduction of opioid-related adverse events using opioid-sparing analgesia with COX-2 inhibitors lacks documentation: a systematic review. Acta Anaesthesiol Scand.

[24] Sanderson BJ, Doane MA (2018). Transversus Abdominis Plane Catheters for Analgesia following abdominal Surgery in adults. Reg Anesth Pain Med.

[25] Tiippana EM (2007). Do surgical patients benefit from perioperative gabapentin/pregabalin? A systematic review of efficacy and safety. Anesth Analg.

[26] Maund E (2011). Paracetamol and selective and non-selective non-steroidal anti-inflammatory Drugs for the reduction in morphine-related side-effects after major Surgery: a systematic review. Br J Anaesth.

[27] Mythen MG (2005). Postoperative gastrointestinal tract dysfunction. Anesth Analg.

[28] Korolkiewicz RP (2003). Differential salutary effects of nonselective and selective COX-2 inhibitors in postoperative ileus in rats. J Surg Res.

[29] Whittle BJ (2003). Gastrointestinal effects of nonsteroidal anti-inflammatory Drugs. Fundam Clin Pharmacol.

[30] Mackway-Jones K (1999). Modification of the cardiovascular response to Hemorrhage by somatic afferent nerve stimulation with special reference to gut and skeletal muscle blood flow. J Trauma.

[31] Grundy D (2002). Neuroanatomy of visceral nociception: vagal and splanchnic afferent. Gut.

[32] Khasar SG (2003). Fasting is a physiological stimulus of vagus-mediated enhancement of nociception in the female rat. Neuroscience.

[33] Amar PJ, Schiff ER (2007). Acetaminophen safety and hepatotoxicity–where do we go from here?. Expert Opin Drug Saf.

[34] Ceelie I (2013). Effect of intravenous Paracetamol on postoperative morphine requirements in neonates and infants undergoing major noncardiac Surgery: a randomized controlled trial. JAMA.

[35] Klein M, Gögenur I, Rosenberg J (2012). Postoperative use of non-steroidal anti-inflammatory Drugs in patients with anastomotic leakage requiring reoperation after colorectal resection: cohort study based on prospective data. BMJ.

[36] Tunali Y (2013). Efficacy of intravenous Paracetamol and dexketoprofen on postoperative pain and morphine consumption after a lumbar disk Surgery. J Neurosurg Anesthesiol.

[37] Zafar N (2010). The evolution of analgesia in an ‘accelerated’ recovery programme for resectional laparoscopic colorectal Surgery with anastomosis. Colorectal Dis.

